# Lack of M_4_ muscarinic receptors in the striatum, thalamus and intergeniculate leaflet alters the biological rhythm of locomotor activity in mice

**DOI:** 10.1007/s00429-020-02082-x

**Published:** 2020-05-14

**Authors:** Vladimir Riljak, Katerina Janisova, Jaromir Myslivecek

**Affiliations:** Institute of Physiology, 1st Faculty of Medicine, Charles University, Albertov 5, 12800 Prague, Czech Republic

**Keywords:** Locomotor activity, Biorhythm, M_4_ muscarinic receptors, M_1_ muscarinic receptors, Intergeniculate leaflet

## Abstract

The deletion of M_4_ muscarinic receptors (MRs) changes biological rhythm parameters in females. Here, we searched for the mechanisms responsible for these changes. We performed biological rhythm analysis in two experiments: in experiment 1, the mice [C57Bl/6NTac (WT) and M_4_ MR −/− mice (KO)] were first exposed to a standard LD regime (12/12-h light/dark cycle) for 8 days and then subsequently exposed to constant darkness (for 24 h/day, DD regime) for another 16 days. In experiment 2, the mice (after the standard LD regime) were exposed to the DD regime and to one light pulse (zeitgeber time 14) on day 9. We also detected M_1_ MRs in brain areas implicated in locomotor biological rhythm regulation. In experiment 1, the biological rhythm activity curves differed: the period (*τ*, duration of diurnal cycle) was shorter in the DD regime. Moreover, the day mean, mesor (midline value), night mean and their difference were higher in KO animals. The time in which the maximal slope occurred was lower in the DD regime than in the LD regime in both WT and KO but was lower in KO than in WT mice. In experiment 2, there were no differences in biological rhythm parameters between WT and KO mice. The densities of M_1_ MRs in the majority of areas implicated in locomotor biological rhythm were low. A significant amount of M_1_ MR was found in the striatum. These results suggest that although core clock output is changed by M_4_ MR deletion, the structures involved in biological rhythm regulation in WT and KO animals are likely the same, and the most important areas are the striatum, thalamus and intergeniculate leaflet.

## Introduction

The generation of the rhythmic pattern controlling locomotion is formed by the activity of intrinsically oscillating interneurons in the spinal cord (Wyartt 2018). Researchers now agree that locomotion is generated centrally in the spinal cord by circuits referred to as central pattern generators (CPGs, see Table 1 for list of abbreviations). CPGs are triggered by descending commands from the brain (initializing or halting). In addition to on-demand triggering of these commands to meet the homeostatic needs of the organism, locomotion itself reveals a diurnal pattern directed by a series of pacemakers. The main circadian pacemaker is localized in the hypothalamic suprachiasmatic nuclei (SCN) (Ballesta et al. 2017). However, some other brain structures have recently been identified as important in locomotor biological rhythm regulation: the subparaventricular zone (SPVZ), intergeniculate leaflet (IGL) (Morin 2013), and posterior hypothalamic area (PHA) (Abrahamson and Moore 2006; Hughes and Piggins 2012). Locomotor activity can also be considered a nonphotic entraining signal of circadian rhythmicity (Hughes and Piggins 2012).

**Table 1:** List of abbreviations

Abbreviation	Explanation
CPGs	Central pattern generators
FFT	Fast Fourier transformation
SCN	Suprachiasmatic nuclei
SPVZ	Subparaventricular zone
IGL	Intergeniculate leaflet
PHA	Posterior hypothalamic area
MR	Muscarinic receptors
WT	Wild types
KO	Knockout animals
LD regime	12/12-h light/dark cycle, 22 ± 1 °C, light on at 7:00 AM
DD regime	Constant darkness (for 24 h/day)
WT1-8	WT animals, 1st to 8th day
WT9-16	WT animals, 9th to 16th day
KO1-8	KO animals, 1st to 8th day
KO9-16	KO animals, 9th to 16th day
*D*_mean_	Day mean
*N*_mean_	Night mean
*N*–*D*_mean_	Difference between night and day mean
mesor	A midline based on the distribution of values across the cycles of the circadian rhythm, computed using a cosine function
*τ*	Period, the time after which a defined phase of the oscillation re-occurs
Periodogram	Lomb-Scargle analysis of the period(s) power spectrum
Actogram	The graphic representation of animal activity expressed by black sections (activity) and white sections (no activity)

SCN has long been considered a structure rich in cholinergic neurons, i.e., nicotinic cholinergic receptors have been identified there by the end of the 70s (Silver and Billiar 1976; Segal et al. 1978). Immunohistochemical study by (van der Zee et al. 1991) has identified colocalization of nicotinic and muscarinic receptors in the SCN. SCN has been shown to be innervated by cholinergic nerves (Hut and Van der Zee 2011) but does not have to be necessarily intrinsically cholinergic (van den Pol and Tsujimoto 1985). It receives cholinergic projections from basal forebrain and brain stem tegmentum (Bina et al. 1993). Another approach, determining especially cholinergic neurons using cholineacetyltransferase positivity, has been used by Ichikawa and Hirata (1986). This study concluded that among other structures, SCN (ventromedial aspect) is rich in cholinergic terminals. No cholinergic terminals have been found in other hypothalamic areas.

The cholinergic agonist carbachol has been shown to have similar effects on the circadian rhythm in rat pineal serotonin *N*-acetyltransferase activity (Zatz 1979; Zatz and Brownstein 1979). It has also been demonstrated that carbachol pellet, implanted near the SCN, shortened the free-running period under constant darkness (Furukawa et al. 1987). However, there are species differences in the presence of cholinergic neurons in the SCN in rat, hamster and mouse (Hut and Van der Zee 2011). For example, carbachol (i.c.v. injection) was able to bring about phase shifts but was not able to induce these effects when injected directly into the SCN (Buchanan and Gillette 2005) in mice while in hamsters the phase shifts were remarkable both after injection into the ventricle or SCN (Bina and Rusak 1996).

Muscarinic receptor subtype expression in the SCN is still a matter of debate. An initial paper used autoradiography with the aim to determine the presence of MR in the SCN (Bina et al. 1998). These authors revealed that MR density in the SCN is very low, especially when compared to the striatum. Further research has identified M_1_ MR (Liu and Gillette 1996) to be present in the SCN. Another report indicated the presence of MR (generally) using immunohistochemistry (Hut and Van der Zee 2011). It is not surprising that PCR technique identified all five MR subtypes in the rat SCN (Yang et al. 2010). This study also determined carbachol inhibitory effects (carbachol hyperpolarization) in the SCN and found that both M_4_ and M_1_ receptors are involved (Yang et al. 2010). Carbachol induced phase advance in the circadian rhythm of spontaneous neuronal activity (Gillette et al. 2001) was assigned to M_1_ MR. Another recent data suggest the role of M_4_ MR in biological rhythm regulation: the M_4_ positive allosteric modulator LY2033298 enhanced oxotremorine (muscarinic agonist) inhibitory effect on light-induced phase delays but had no effect by itself (Gannon and Millan 2012).

The role of neurotransmitter systems in circadian rhythms has been reviewed by Rusak and Bina (1990). In addition, biological rhythms of many receptors, including muscarinic receptors, have been identified in the whole forebrain and the striatum that varied during the year and in various light conditions (Kafka et al. 1983; Wirz-Justice 1987).

Not only SCN but also other structures are involved in light-like effects. Thus, it has been shown that extra-SCN cholinergic synapse mediates the light-like cholinergic clock resetting reported previously (Buchanan and Gillette 2005). Another way how to induce phase shift is muscarinic input into the IGL (Cain et al. 2007). Moreover, there could be a fine-tuned balance between cholinergic and glutamatergic neurons in the IGL, as has been shown by Pekala et al. (2007). These authors have demonstrated that in the presence of a cholinergic agonist, glutamate-induced activity was either decreased or increased or not changed.

M_4_ muscarinic receptors (MRs) have been suggested to play a role in motor coordination. Previous studies have shown different results depending on genetic background and number of backcrosses (Fink-Jensen et al. 2011; Gomeza et al. 1999; Koshimizu et al. 2012; Schmidt et al. 2011; Woolley et al. 2009). However, no attention has been given to biological rhythms. In our recent work (Valuskova et al. 2018b), we analyzed telemetrically obtained biological rhythms under a light/dark cycle (LD 12/12 h, lights on at 6:00) in intact M_4_KO mice (activity, body temperature) grown on the C57Bl6 background (i.e. the mice were backcrossed to C57Bl6 line for 12 generations) using ChronosFit software. We showed that M_4_KO female mice motor activity did not differ substantially from that of wild-type mice during the light period, while in the dark phase (the active part of the day for mice), the M_4_KO mice revealed an increase in the mesor, in the night values, in the night-day difference, in the area under curve, in the highest value, in the night area under curve, in the highest value measured during the night period, and in the amplitudes of the 24-h, 12-h, 6-h, and 4.8-h rhythm. Similar differences have also been found between female and male KO mice.

As the brain areas implicated in locomotor activity biological rhythm changes comprise more structures (see also above), we employed in vitro autoradiography and identified potential brain areas likely involved in locomotor activity biological rhythm regulation. We investigated the following areas: the motor cortex, the striatum, the thalamus, and the intergeniculate leaflet. M_4_ MR expression was negligible in the subparaventricular zone, the posterior hypothalamic area, and the suprachiasmatic nuclei (Valuskova et al. 2018b).

In subsequent work (Valuskova et al. 2019), we also identified important differences in the morning vs. evening muscarinic drug (scopolamine, oxotremorine) effects, both in WT and M_4_ KO animals. Acutely, scopolamine induced an increase in motor activity in WT and M_4_KO at 9:00, yet no significant increase was observed at 21:00. Oxotremorine induced hypothermic effects in both WT and M_4_KO. Hypothermic effects were more evident in WT than in M_4_KO. Hypothermia in both cases was more pronounced at 9:00 than at 21:00. We have also tested cocaine as a drug that can disrupt the balance between dopamine and acetylcholine levels. Cocaine increased motor activity when compared to saline, but no differences were found in morning vs. evening effects. Another part of our study focused on behavior in a novel environment. There was no difference in behavior in the open field between WT and M_4_KO when tested at 9:00; however, at 21:00, the activity of M_4_KO mice was doubled in comparison to that of WT mice. Both WT and KO animals spent less time climbing in their active phase. Autoradiography of muscarinic receptors, GABA_A_ receptors, dopamine D_1_-like receptors, D_2_-like receptors, kainate receptors and NMDA receptors revealed no significant morning *vs.* evening differences.

Here, we searched for a potential mechanism of locomotor biological rhythm changes using constant darkness to distinguish light responsiveness from real circadian effects. We tested the hypothesis that the increased locomotion in females is caused by circadian rhythmicity changes. In addition, we also identified specific structures involved in locomotor biological rhythm changes. We used a light pulse to cause a phase shift. We tested the hypotheses that the phase shift would reveal similar parameters in WT and KO animals, thus suggesting that similar CNS structures are involved in M_4_ MR-affected changes in biological rhythm. As there were evident differences between brain areas in M_4_ MRs density, we also investigated another MRs subtype that is believed to be involved in motor coordination—the M_1_ MRs.

## Methods

### Animals

Mice lacking the M_4_ muscarinic receptor were generated in Wess’ laboratory (Gomeza et al. 1999) and then bred in our animal facility (Prague, Czech Republic). Their genetic background was C57Bl/6NTac. Animals were treated in accordance with the legislature of the Czech Republic and the EU legislature (European Convention for the Protection of Vertebrate Animals used for Experimental and other Scientific Purposes [Council of Europe N^o^ 123, Strasbourg 1985]), and the experimental protocol was approved by the Committee for the Protection of Experimental Animals of the 1st Medical Faculty, Charles University, Prague and by the Ministry of Education of the Czech Republic under N^o^ MSMT-2409/2017-3. The wild-type line was the C57Bl/6NTac line. We studied fully backcrossed (14 generations) muscarinic M_4_^−/−^ and M_4_^+/+^ littermates. The animals were maintained under controlled environmental conditions (12/12-h light/dark cycle, 22 ± 1 °C, light on at 7:00). Food and water were available ad libitum. A total of 36 females (weighing 20–26 g, age 3–6 months) were used in the study, of which there were 18 M_4_ KO animals and 18 WT animals. Prior to the experiments, the mice were genotyped, and only homozygous mice were used. The females were housed separately from males, thus revealing the Lee-Boot effect (i.e., suppression of the estrus cycle–anestrous), which made the female group homogenous in hormone levels. Moreover, no differences were observed in light microscopy of vaginal lavage or actograms in females for 15 consecutive days (control animals, not included in the experiment because of the stressful procedure associated with lavage acquisition).

### Telemetry

To judge the biological rhythm changes, we employed a telemetric apparatus able to measure body temperature and overall motor activity. The telemetry system used was commercially available from Mini Mitter (Starr Life Sciences Corp., Oakmont, PA, USA, originally from Respironics, Andover, MA, USA). The transponders (E-Mitter, G2, length 15.5 mm, 1.1 g) were implanted in the peritoneal cavity under anesthesia (Zoletil^®^ 100, Rometar^®^ 2% 5:1, diluted 10 times, 3.2 ml kg^−1^). During the implantation, the mice were kept on a thermostable pad. Mice were allowed 2 weeks for recovery from the surgery and then used in the experiment. The activity data was acquired directly from the transponders in the sample period for three consecutive days, during which the animals were not disturbed. Similar rhythms were recorded before and after this sample period. The activity was recorded in home cages of typical size (38 × 22 × 15 cm). Receivers were connected in series and connected directly to the PC into a single computer port, allowing for the determination of all parameters. The data were collected every 60 s. VitalView software was used for the acquisition and first processing of data.

### Biological rhythm analysis

The data collected by telemetry were grouped into 10-min sequences, and the calculated means were used for further analysis. The analysis was performed by Lomb-Scargle spectral analysis (period length and power spectra determination), by fast Fourier transformation (FFT) and the stepwise regression technique as described earlier (Valuskova et al. 2018b) with the determination of biological rhythm parameters. Phase shifts of activity were calculated by determining the horizontal distance between regression lines fitted through activity onsets and offsets in the LD and DD regimes. The times of the minimal and maximal slopes were compared between control (WT) and experimental (M_4_ KO) groups in the LD and DD regimes. The data were also presented as periodograms and actograms. For further statistical analysis GraphPad Prism 8. 4.0.0 program (San Diego, USA) was used.

### Autoradiography detection of M_1_ muscarinic receptors

For receptor determination, autoradiography was performed in several brain areas previously shown to be connected with locomotor biological rhythm changes (striatum [Str], thalamus [TH], SCN, SPVZ, PHA, and IGL). Brains were rapidly removed (4–6 brains per group), frozen in dry ice, and then stored at − 80 °C until cryostat sectioning. Sixteen-micrometer-thick sagittal or frontal sections were cut on a cryostat at − 20 °C, thaw-mounted on Superfrost^®^ Plus glass slides (Carl Roth GmbH & Co. KG, Karlsruhe, Germany) and stored in storage boxes at − 80 °C until use. To assess M_1_ muscarinic receptor binding, the sections were allowed to thaw and dry for 30 min at 22 °C, and the density of receptors was determined using the M_1_ MR selective protocol as previously described (Valuskova et al. 2018a). Briefly, sections were incubated for 1 h with 5 nM ^3^H-pirenzepine at room temperature. Nonspecific binding was assessed on adjacent sections in the presence of 10 µM atropine sulfate. After incubation, the sections were washed two times for 5 min and gently dried. Dry sections were apposed to the tritium-sensitive Fuji BAS imaging plates (GE Healthcare Europe GmbH, Freiburg, Germany) in Kodak Biomax autoradiographic cassettes (Carestream Health, Inc., Rochester, NY, USA) for 5 days. The linearity of the signal and conversion of photostimulated luminescence to radioactivity was assessed using tritium autoradiographic standards (American Radiolabeled Chemicals, Inc., St. Louis, MO, USA). The film autoradiograms were scanned, and densitometry was performed with the PC-based analytical software, MCID analysis software. Measurements were taken and averaged from at least three sections for each animal and brain region. We compared the densities in the left and right hemisphere. Since there were no differences in laterality, both sides were taken together.

### Histology

Nissl staining was used for SCN, SPVZ, IGL and PHA identification in MR autoradiography determination. Briefly, parallel sections were obtained using a cryostat [the appropriateness of the section was assessed using a mouse atlas (Paxinos and Franklin 2008)], and the sections were collected and divided into four sets. The first section from the set was placed on the first glass slide and used for Nissl staining, while the remaining four sections from the set were placed on other glass slides (three sections from different sets on one glass slide) and used for autoradiography. The sections used for Nissl staining were immersed in a solution of alcohol (70%, 80%, 96%) for two minutes each, stained with Nissl solution (1% cresyl violet and 0.2 mol/l acetatic acid + 0.2 mol/l sodium acetate, 4:1, pH = 3) for 20 min, then twice washed in distilled water and immersed in a solution of alcohol (96%, 80%, 70%) for two minutes each. Then, the samples were immersed in xylen (xylen, mixture of isomers, p.a., Penta, Czech Republic) for 5 min. Then, the sections were incubated for another 45 min in xylen (p.a., Penta, Czech Republic) and mounted using DPX (SigmaAldrich, Czech Republic) with a coverslip.

The area, clearly visible with Nissl staining, was then marked (using border transposition) on a scanned autoradiogram and used for densitometry with PC-based analytical software (MCID software).

### Experiment 1

The mice were first exposed to a standard LD regime (the same as described above, i.e., 12/12-h light/dark cycle, light on at 7:00) for eight consecutive days and then exposed to constant darkness (for 24 h/day, DD regime) for the other sixteen days. The biological rhythm was analyzed using the FFT and Lomb-Scargle spectral analysis. Lomb-Scargle periodograms were computed and power spectrum in WT and M_4_ KO animals was compared using repeated measures two way-ANOVA with Sidak post hoc correction.

### Experiment 2

The mice were first exposed to a standard LD regime (the same as described above, i.e., 12/12-h light/dark cycle, light on at 7:00) for eight consecutive days and then exposed to constant darkness (for 24 h/day, DD regime) for the other sixteen days. On day 9 from the beginning of the experiment, the animals were exposed to one light pulse (300 lx, 1 h, administered at zeitgeber time 14, the onset of subjective night). The biological rhythm was analyzed using the FFTand Lomb-Scargle spectral analysis.. Lomb-Scargle periodograms were computed and power spectrum in WT and M_4_ KO animals was compared using repeated measures two way-ANOVA with Sidak post hoc correction.

### Statistical analysis

Repeated-measures three-way ANOVA was used for comparison of data in biological rhythm curves. Repeated-measures two-way ANOVA and its alternative mixed-effects model (Restricted Maximum Likelihood, REML) with Sidak post hoc correction were used for specific biological rhythm parameter changes between days 1–8 (LD regime), 9–16 and 17–24 (DD regime). The data reported for REML analysis are similar to ANOVA, except *η*^2^, as the mixed-effects model compares the fit of a model where subjects are a random factor vs. a model that ignores the difference between subjects. This results in *χ*^2^ ratio and *p* value. For comparison of time shifts, an unpaired *t* test was used. For M_1_ MR autoradiography analysis, two-way ANOVA with Sidak post hoc correction was used. Generally, values of *p* < 0.05 were considered significant. The specific test that was used and the factor significance and/or interaction significance are presented in the Results section. The statistics were calculated using GrapPad Prism 8.0.4.0.

## Results

### Experiment 1

#### Biological rhythm of locomotor activity

Lomb-Scargle spectral analysis showed differences between days 1–8 of the experiment (LD regime) and 9–16 (DD regime) both in WT and KO animals in the period length. REML analysis (*χ*^2^ ratio = 5.301, *df* = 1, *p* = 0.0213, main effect of time: *F*_1.171,32.79_ = 12.23, *p* = 0.0008 (*F*_DFn, DFd_, where DFn is the degrees of freedom numerator, and DFd is the degrees of freedom denominator; no effect of genotype: *F*_1,34_ = 0.027, *p* = 0.87 and significant genotype-time interaction: *F*_2,56_ = 12.72, *p* < 0.0001) showed a shortening of the period length in WT and KO animals between days 1–8 (LD regime) and days 9–16 (DD regime). The values were: day 1–8: 24.21 ± 0.023 vs. 23.91 ± 0.016, day 9–16: 23.78 ± 0.06 vs. 23.72 ± 0.02, day 17–24: 23.7 ± 0.11 vs. 24.03 ± 0.12, in WT and KO, respectively.

**Fig. 1:**
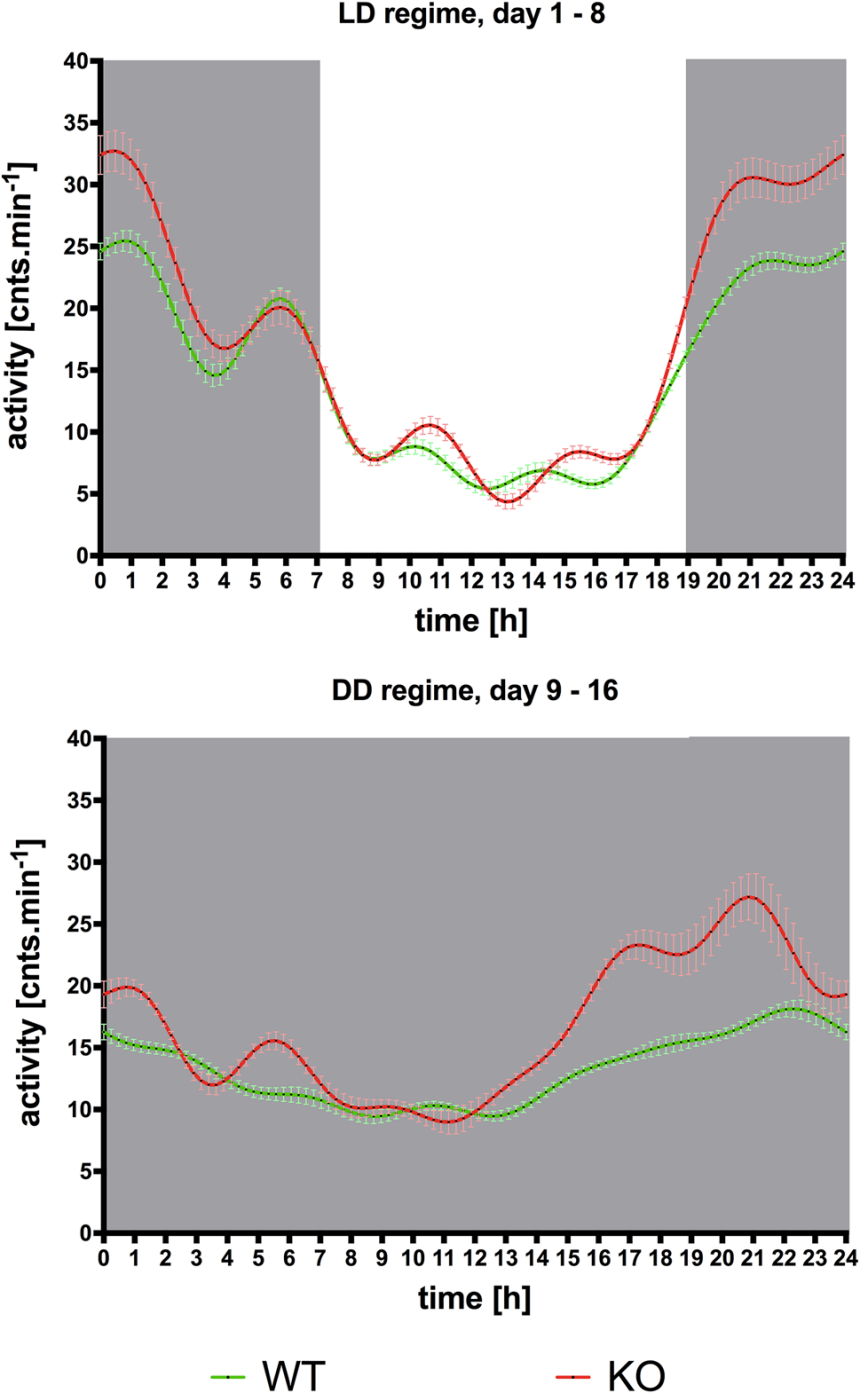
The locomotor activity biological rhythm in the LD regime (days 1–8), top, and in the DD regime (days 9–24), bottom. Gray parts represent the dark phase. Abscissa: time (h), ordinate: activity (cnts.min^−1^). For significance, see “Results”. See legend for symbol explanation

The biological rhythm of locomotor activity, as revealed by FFT, differed between WT and KO animals and between days 1–8 of the experiment (LD regime) and days 9–16 (DD regime, see Fig. 1; repeated-measures three-way ANOVA, genotype: wild-type/knockout; day: days 1–8 (LD regime)/days 9–16 (DD regime); time: time in 24-h cycle from FFT calculation), with a significant main effect of the genotype, time, and day and an interaction of genotype and time (genotype: *F*_1,1400_ = 319.7, *p* < 0.0001; *η*^2^ = 0.016; day: *F*_1,1400_ = 1144, *p* < 0.0001, *η*^2^ = 0.07; time: *F*_99,1400_ = 93.43, *p* < 0.0001 *η*^2^ = 0.57; genotype–time interaction: *F*_99,1400_ = 19.54, *p* < 0.0001, *η*^2^ = 0.096; interaction time–day: *F*_99,1400_ = 13.33, *p* < 0.0001, *η*^2^ = 0.08; genotype–day interaction: *F*_1,1400_ = 10.18, *p* = 0.0014, *η*^2^ < 0.0001; interaction time–genotype–day: *F*_99,1400_ = 3.397, *p* < 0.0001, *η*^2^ = 0.017). To further analyze and identify the significance of differences between WT/KO and days 1–8/9–16, we simplified the analysis design (as the main effect of genotype and day and its interaction was highly significant in the three-way ANOVA, we used WT1-8, KO1-8, WT9-16, and KO9-16 as separate groups without distinguishing genotype and day) and used repeated-measures two-way ANOVA with post hoc Sidak corrections. This analysis (group: wild-type days 1–8/knockout days 1–8/wild-type days 9–16/knockout days 9–16; time: time in a 24-h cycle from the FFT calculation) showed the following: significant main effect of time (*F*_2.378,1665_ = 582.4, *p* < 0.0001, *η*^2^ = 0.086) and group (*F*_700,2100_ = 1.479, *p* < 0.0001, *η*^2^ = 0.051) and an interaction between group and time (*F*_297,2100_ = 13.16, *p* < 0.0001, *η*^2^ = 0.19). Post hoc Sidak analysis showed differences between every group (*p* < 0.0001).

#### Biological rhythm parameters

**Fig. 2:**
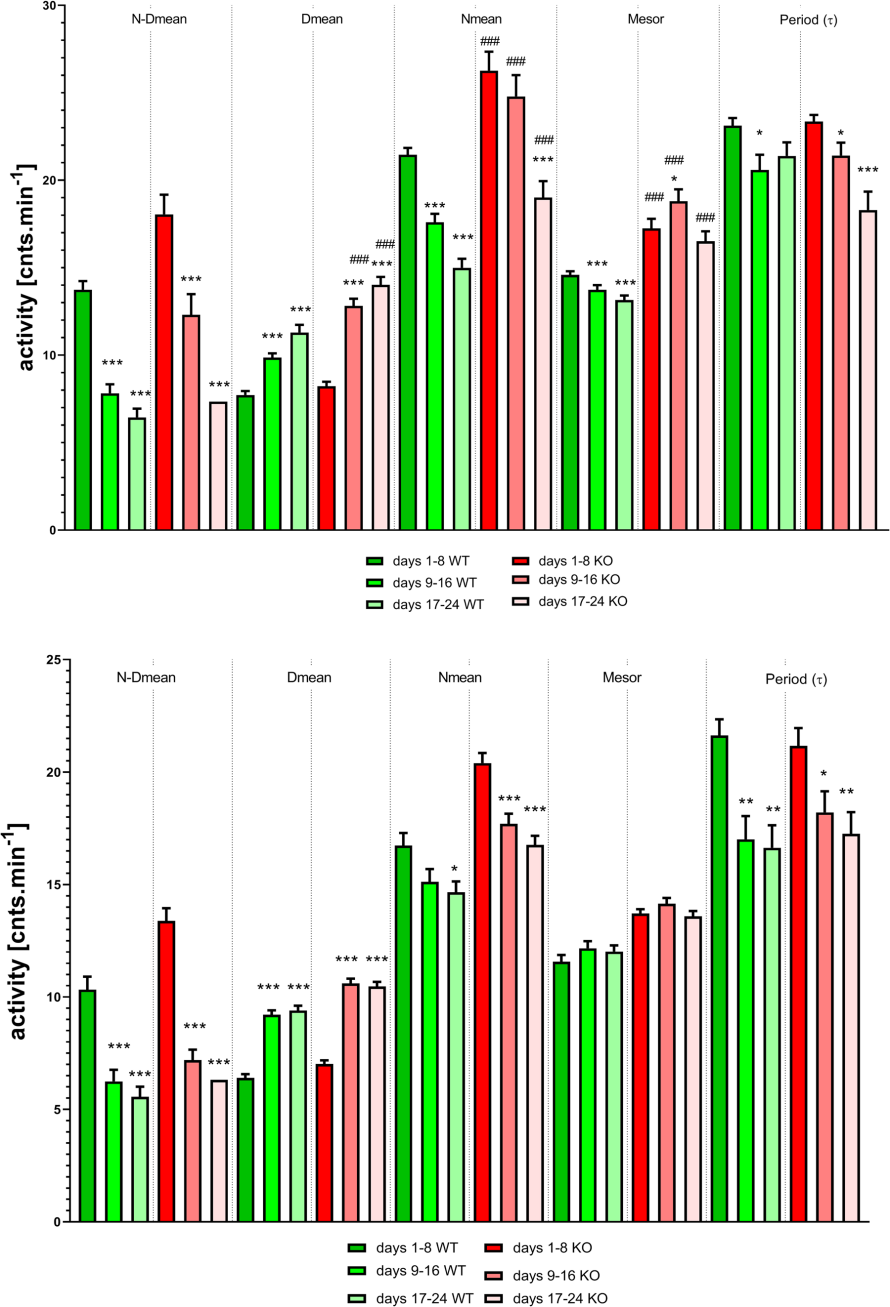
Top: Changes in biological rhythm parameters, *N*–*D*_mean_, *D*_mean_, *N*_mean_, mesor and period (*τ*), before and after the shift from the LD to DD regime. *p < 0.05, difference from the LD regime (days 1–8); ***p < 0.001, difference from the LD regime (days 1–8); ^###^*p* < 0.001, difference from the same time slot in WT animals. See legend for symbol explanation. Bottom: Changes in biological rhythm parameters, *N*–*D*_mean_, *D*_mean_, *N*_mean_, mesor and period (*τ*), before and after the shift from the LD to DD regime with a light pulse (300 lx) applied on day 9. *p < 0.05, difference from the LD regime (days 1–8); **p < 0.01, difference from the LD regime (days 1–8); ***p < 0.001, difference from the LD regime (days 1–8); no difference between KO and WT animals was found

Thus, we compared the main biological rhythm parameters using the mixed-effects model (REML) with Sidak correction. We compared the following parameters on days 1–8 (LD regime), 9–16 (DD regime), and 17–24 (DD regime): day mean (*D*_mean_), night mean (*N*_mean_), difference between night and day mean (*N*–*D*_mean_), mesor (Midline Estimating Statistic of Rhythm, a midline based on the distribution of values across the cycles of the circadian rhythm, computed using a cosine function by fitting the partial Fourier series) and period (*τ*) computed by the FFT (see Fig. 2 top). The switch to the DD regime caused (REML analysis, *χ*^2^ ratio = 5.399, *df* = 1, *p* = 0.0201, main effect of time: *F*_1.857,223.7_ = 13.46, *p* < 0.001; main effect of genotype: *F*_1,137_ = 0.97, *p* = 0.33 and significant genotype-time interaction: *F*_2,241_ = 4.375, *p* = 0.014) a shortening of the period from *τ* = 23 ± 0.44 on days 1–8 to *τ* = 21 ± 0.87 on days 9–16 (*p* = 0.02) in WT animals. In KO animals, the period shortened from *τ* = 23 ± 0.38 on days 1–8 to *τ* = 21 ± 0.74 on days 9–16 (*p* = 0.029) and to *τ* = 18 ± 1.0 (*p* < 0.001) on days 17–24.

*N*–*D*_mean_ REML analysis (*χ*^2^ ratio = 55.32, *df* = 1, *p* < 0.0001) showed the following results: a significant main effect of time (*F*_1.806,246.5_ = 130.5, *p* < 0.001) and genotype (*F*_1,140_ = 11.05, *p* < 0.01) but no significant interaction between genotype and time (*F*_2,273_ = 2.887). Post hoc Sidak analysis was, therefore, performed to assess the time effects in the WT and KO groups separately. This analysis showed differences between time slots in WT animals (*p* < 0.001), i.e., between days 1–8 and 9–16 and between days 1–8 and days 17–24. The same was true for KO animals. *D*_mean_ REML analysis (*χ*^2^ ratio = 16.47, *df* = 1, *p* < 0.0001) showed the following results: a significant main effect of time (*F*_1.701,232.2_ = 116.2, *p* < 0.001) and genotype (*F*_1,140_ = 35.12, *p* < 0.001) and a significant interaction between genotype and time (*F*_2,273_ = 8.88, *p* < 0.001). Post hoc Sidak analysis showed differences between time slots in WT and KO animals (*p* < 0.001) and differences between the WT and KO groups on days 9–16 and 17–24 (*p* < 0.001). REML analysis showed differences in the Nmean (*χ*^2^ ratio = 126.9, *df* = 1, *p* < 0.0001) including a significant main effect of time (*F*_1.905,260_ = 76.3, *p* < 0.001) and genotype (*F*_1,140_ = 27.81, *p* < 0.001) and a significant interaction between genotype and time (*F*_2,273_ = 4.211, *p* = 0.016). Post hoc Sidak analysis showed differences between time slots in WT animals (*p* < 0.001) and between days 1–8 and 17–24 in KO animals, as well as a significant difference between WT and KO on days 1–8, 9–16 and 17–24 (*p* < 0.001). REML analysis also showed differences in the mesor (*χ*^2^ ratio = 167.1, *df* = 1, *p* < 0.0001) including a significant main effect of time (*F*_1.987,271.3_ = 14.25, *p* < 0.001) and genotype (*F*_1,140_ = 41.56, *p* < 0.001) and a significant interaction between genotype and time (*F*_2,273_ = 9.26, *p* < 0.001). Post hoc Sidak analysis showed differences between time slots in WT animals (*p* < 0.001) and between days 1–8 in KO animals, as well as a significant difference between the WT and KO groups on days 1–8, 9–16 and 17–24 (*p* < 0.001).

**Fig. 3:**
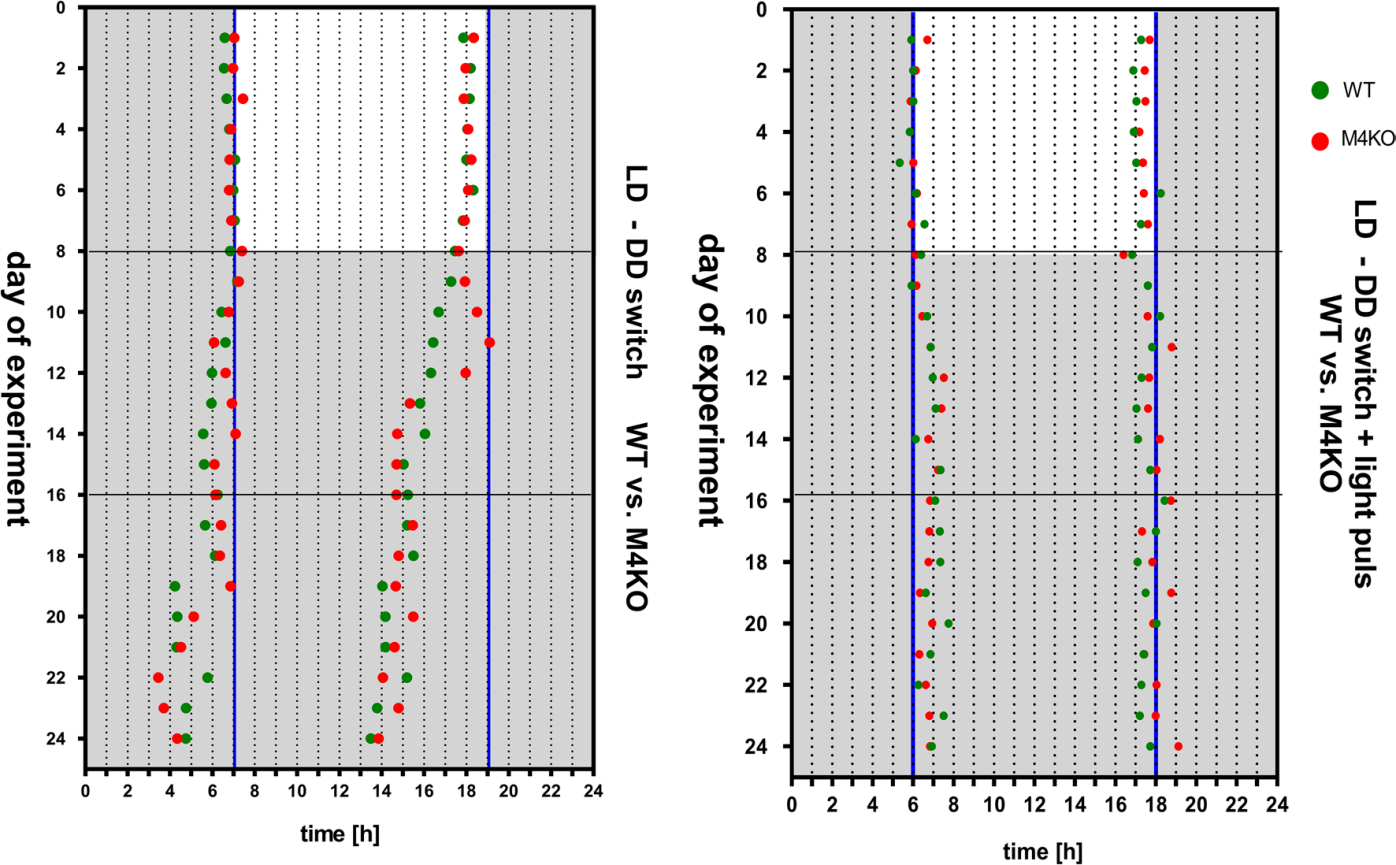
Left: Timeshift after the switch to the DD regime expressed as *t*_MinSlope_ and *t*_MaxSlope_ in WT and KO animals. Horizontal lines represent division of time slots (days 1–8, 9–16, 17–24). See legend for symbol explanation. For significance, see “Results”. Right: time shift after switching to the DD regime with light pulse (300 lx) applied on day 9, expressed as *t*_MinSlope_ and *t*_MaxSlope_ in WT and KO animals. Horizontal lines represent the division of time slots (days 1–8, 9–16, 17–24). See legend for symbol explanation. For significance, see “Results”

Another aspect of the activity shift is depicted in Fig. 3 left, where the times at which the slope was maximal or minimal are shown. REML analysis of *t*_MinSlope_ (*χ*^2^ ratio = 1.096, *df* = 1, *p* = 0.29) showed that the *p* value is high thus we have to conclude that the matching was not effective. REML analysis returned a significant main effect of time (*F*_1.941,233.9_ = 5.746, *p* = 0.004), whereas the main effect of genotype (*F*_1,137_ = 0.04, *p* = 0.84) and interaction between genotype and time (*F*_2,241_ = 0.1, *p* = 0.9) were not significant. It is, therefore, possible to conclude that there was no difference in *t*_MinSlope_ between WT and KO in LD and DD regime. REML analysis also showed a high *p* value in *t*_MaxSlope_ (*χ*^2^ ratio = 0.017, *df* = 1, *p* = 0.896) but returned a significant main effect of time (*F*_1.911,230.3_ = 21.5, *p* < 0.001) and genotype (*F*_1,137_ = 14.28, *p* < 0.001) and a significant interaction between genotype and time (*F*_2,241_ = 3.3, *p* = 0.029). Post hoc Sidak analysis showed differences between time slots in WT (*p* < 0.001 and *p* < 0.01, between days 1–8 and 9–16 and between days 1–8 and 17–24, respectively) and KO (*p* = 0.038 between days 1–8 and 9–16, and *p* < 0.001 between days 1–8 and 17–24) animals and a difference between the WT and KO groups on days 1–8 (*p* < 0.001) and days 17–24. Taken together, the differences in *t*_MaxSlope_ between WT and KO in LD and DD regime are questionable.

**Fig. 4:**
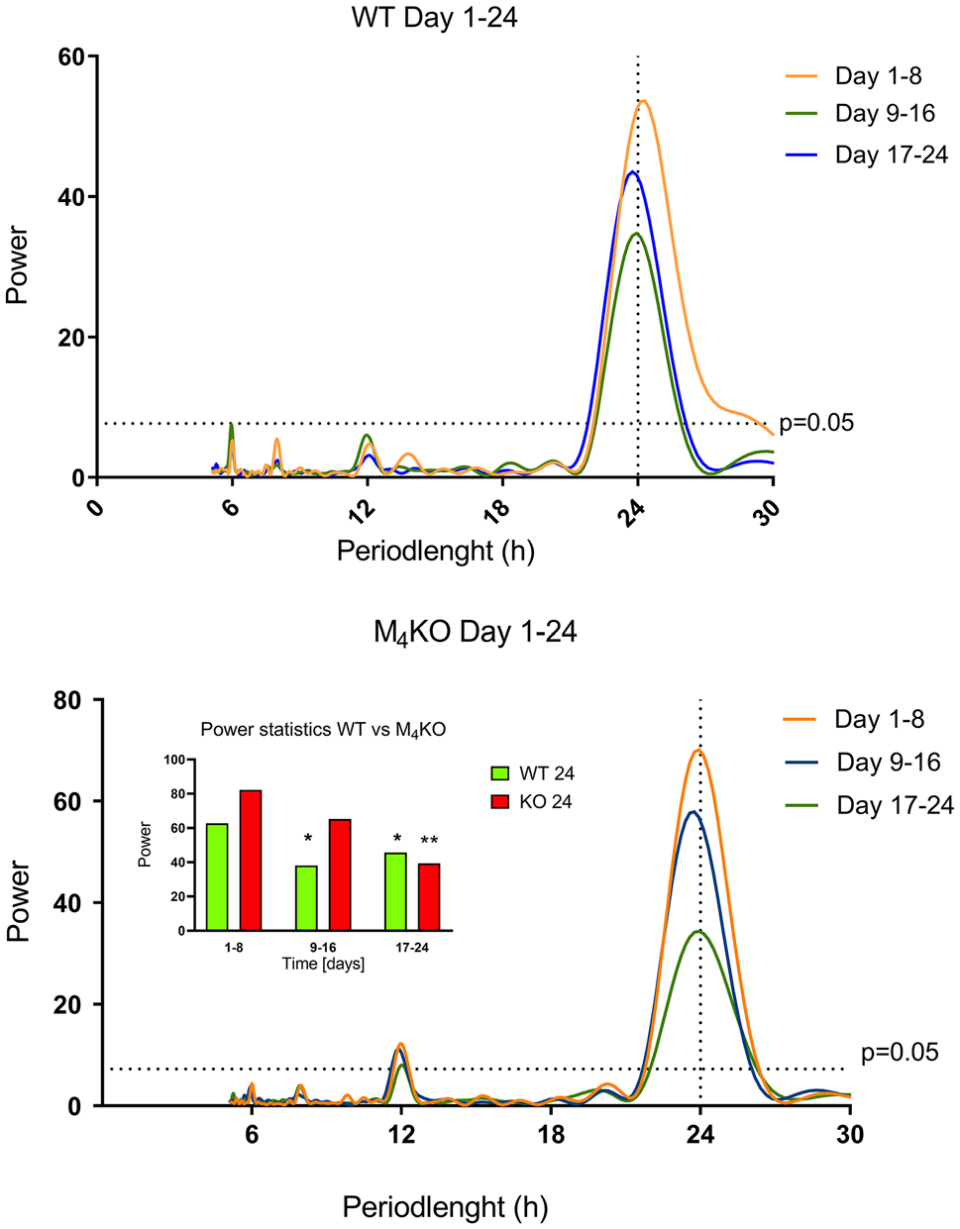
Lomb-Scargle periodograms in WT (top) and M_4_ KO (bottom) animals before (i.e. in LD regime) and after switch to the DD regime. The horizontal dotted line shows the power with *p* < 0.05, the vertical dotted line shows the 24-h period. See legend for symbol explanation. The inset in the M_4_ KO periodogram shows the differences in power in WT and KO animals between specific time slots and the 1–8th day (LD regime). *p < 0.05, difference from the LD regime; **p < 0.01, difference from the LD regime

Lomb-Scargle periodograms (see Fig. 4) showed an approximately 24-h period both in WT and KO animals. Moreover, it was possible to detect an additional, approximately 12-h period in KO animals (day 1–8: 11.93 ± 0.039, day 9–16: 11.87 ± 0.046, day 17–24: 12.03 ± 0.03). The calculation of power spectrum in the 24-h period showed (repeated measures two-way ANOVA, genotype: wild-type and knockout, time: days 1–8, 9–16, 17–24) a significant main effect of time (*F*_1.626, 24.39_ = 26.34, *p* < 0.0001, *η*^2^ = 0.23) and of interaction genotype/time (*F*_2,30_ = 8.595, *p* = 0.0011, *η*^2^ = 0.07). The effect of genotype was not significant (*F*_1,15_ = 1.897, *p* = 0.19, *η*^2^ = 0.06). Post hoc Sidak analysis showed differences between days 1–8 and 9–16, and 1–8 and 17–24 in WT, and between days 1–8 and 17–24 in KO.

The sample periodograms showing the time shift are shown in Fig. 6 (top).

### Experiment 2

#### Biological rhythm parameters

Lomb-Scargle spectral analysis showed no difference between WT and KO animals (REML analysis (*χ*^2^ ratio = 4.425, *df* = 1, *p* = 0.0354) showed no effect of time: *F*_1.901,24.71_ = 2.117, *p* = 0.14; no effect of genotype: *F*_1,15_ = 0.2127, *p* = 0.65 and a significant genotype-time interaction: *F*_2,26_ = 3.733, *p* = 0.0376). Thus no difference in the period length in WT and KO animals between days 1–8 (LD regime) and days 9–16 (DD regime) was revealed. The differences were: day 1–8: 24.04 ± 0.06 vs. 23.91 ± 0.02, day 9–16: 24.05 ± 0.09 vs. 24.13 ± 0.04 and day 17–24: 23.96 ± 0.12 vs. 24.1 ± 0.04, in WT and KO, respectively.

The comparison of the main biological rhythm parameters was performed using FFT analysis with stepwise regression technique and statistically analysed by the mixed-effects model (REML) with Sidak correction. We compared the same parameters in the same time slots as in Experiment 1 (see Fig. 2 bottom). Using this analysis, the light pulse caused (REML analysis, *χ*^2^ ratio = 4.706, *df* = 1, *p* = 0.0301, main effect of time: *F*_1.952,239.1_ = 15.94, *p* < 0.001; main effect of genotype was not significant: *F*_1,141_ = 0.26, *p* = 0.61; genotype-time interaction was not significant: *F*_2,245_ = 0.53, *p* = 0.59) a shortening of the period from *τ* = 21.64 ± 0.71 on days 1–8 to *τ* = 17.01 ± 1.04 on days 9–16 (*p* = 0.002) and to *τ* = 16.64 ± 1.00 on days 17–24 (*p* = 0.002) in WT animals. In KO animals, the period was shortened from *τ* = 21.17 ± 0.79 on days 1–8 to *τ* = 18.21 ± 0.94 on days 9–16 (*p* = 0.04) and to *τ* = 17.26 ± 0.96 (*p* = 0.008) on days 17–24.

Other parameters [*N*–*D*_mean_ (*χ*^2^ ratio = 0.045, *df* = 1, *p* = 0.83), *D*_mean_ (*χ*^2^ ratio = 0.99, *df* = 1, *p* = 0.75), *N*_mean_ (*χ*^2^ ratio = 1.495, *df* = 1, *p* = 0.221), mesor (*χ*^2^ ratio = 1.624, *df* = 1, *p* = 0.20), *t*_MinSlope_ (*χ*^2^ ratio = 0.8, *df* = 1, *p* = 0.37), *t*_MaxSlope_ (*χ*^2^ ratio not estimated)] had very high *p* values. Thus, we have to conclude that the matching was not effective. In spite of that, the analysis returned some significant differences: *N*–*D*_mean_: a significant main effect of time (*F*_1.742,221.3_ = 73.84, *p* < 0.001) and genotype (*F*_1,128_ = 7.25, *p* = 0.008) and no significant interaction between genotype and time (*F*_2,254_ = 2.988, *p* = 0.052). Post hoc Sidak analysis was, therefore, performed to assess the time effects in the WT and KO groups separately. This analysis showed differences between time slots in WT animals (*p* < 0.001), i.e., between days 1–8 and 9–16 and between days 1–8 and days 17–24. The same was true also for KO animals.

*D*_mean_ REML analysis showed the following results: a significant main effect of time (*F*_1.961,276.5_ = 184.2, *p* < 0.001) and genotype (*F*_1,142_ = 40.34, *p* < 0.001), but the genotype-time interaction was not significant (*F*_2,282_ = 2.05, *p* = 0.13). Post hoc Sidak analysis showed differences between time slots (days 1–8 vs. 9–16, and 1–8 vs. 17–24) in WT and KO animals (*p* < 0.001). REML analysis showed differences in *N*_mean_: a significant main effect of time (*F*_1.842,259.8_ = 19.56, *p* < 0.001) and genotype (*F*_1,142_ = 43.08, *p* < 0.001), but the interaction between genotype and time was not significant (*F*_2,282_ = 1.4, *p* = 0.25). Post hoc Sidak analysis showed differences between days 1–8 and 17–24 in WT animals (*p* = 0.028) and between days 1–8 and 9–16 (*p* < 0.001) and days 1–8 and 17–24 (*p* < 0.001) in KO animals. REML analysis also showed a significant main effect of genotype in the mesor (*F*_1,142_ = 67.82, *p* < 0.001). Other parameters were not significant: main effect of time (*F*_1.936,273_ = 2.142, *p* = 0.12) and genotype-time interaction (*F*_2,282_ = 0.66, *p* = 0.52). However, in the view of non-effective matching these differences are questionable.

Another aspect of the activity shift is depicted in Fig. 3 right, where the times in which the slope was maximal or minimal are shown. REML analysis showed differences in *t*_MinSlope_: a significant main effect of time (*F*_1.921,235.3_ = 31.33, *p* < 0.001) and genotype (*F*_1,141_ = 6.673, *p* = 0.01) and a significant genotype–time interaction (*F*_2,245_ = 4.008, *p* = 0.019). Post hoc Sidak analysis showed differences between time slots in WT (*p* < 0.001) and KO animals (*p* < 0.001 between days 1–8 and 9–16, and *p* = 0.013 between days 1–8 and 17–24, respectively). The difference between WT and KO animals was found on days 17–24 (*p* = 0.01). REML analysis also showed differences in *t*_MaxSlope_: a significant genotype–time interaction (*F*_2,386_ = 3.044, *p* = 0.048). However, the main effects were not significant: time (*F*_1.86,359_ = 2.286, *p* = 0.11) and genotype (*F*_1,386_ = 2.833, *p* = 0.11). However, in the view of non-effective matching these differences are questionable.

Time shifts between WT and KO animals were not significantly different between days 1–8 and days 9–16 (Δ*ϕ*_WT_ = 1.08 ± 0.12, (Δ*ϕ*_KO_ = − 0.83 ± 1.58, *t* test, *p* = 0.35) and between days 1–8 and 17–24 (Δ*ϕ*_WT_ = 0.55 ± 0.95, (Δ*ϕ*_KO_ = − 0.73 ± 1.34, *t* test, *p* = 0.52).

**Fig. 5:**
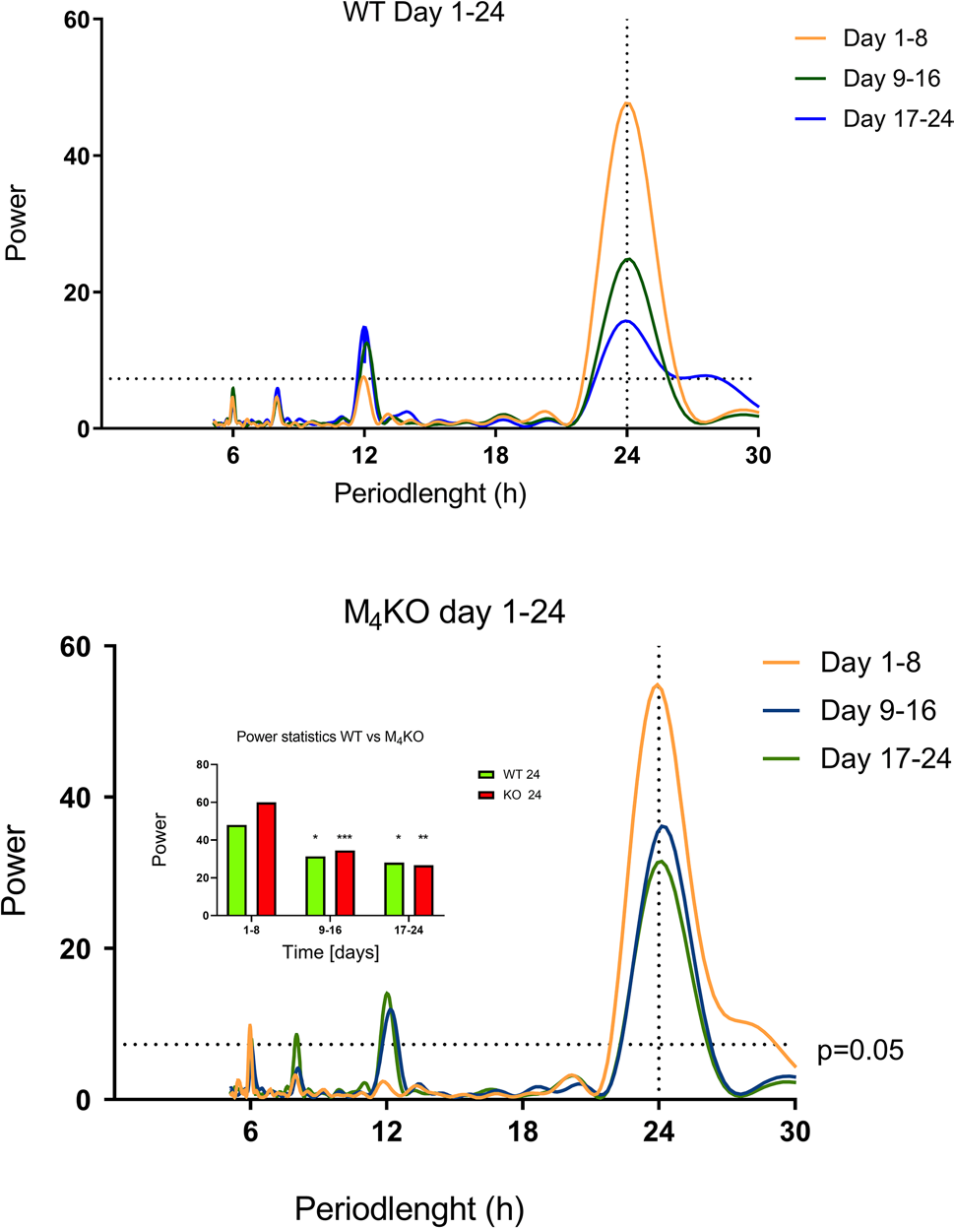
Lomb-Scargle periodograms in WT (top) and M_4_ KO (bottom) animals before (i.e. in LD regime) and after the switch to the DD regime when the light pulse was applied. The horizontal dotted line shows the power with *p* < 0.05, the vertical dotted line shows the 24-h period. See legend for symbol explanation. The inset in the M_4_ KO periodogram shows the differences in power in WT and KO animals between specific time slots and the 1–8th day (LD regime). *p < 0.05, difference from the LD regime; **p < 0.01, difference from the LD regime

Lomb-Scargle periodograms showed (see Fig. 5) an approximately 24-h period both in WT and KO animals. Moreover, it was possible to detect an additional, approximately 12-h period in WT and KO animals but the differences between WT and KO were non-significant (day 9–16: WT: 12.13 ± 0.04, KO: 12.14 ± 0.06, day 17–24: WT: 12.03 ± 0.02, KO: 12.04 ± 0.04). The calculation of power spectrum in the 24-h period showed [mixed-effects model (REML) with Sidak correction, genotype: wild-type and knockout, time: days 1–8, 9–16, 17–24] a significant main effect of time (*F*_1.526,19.84_ = 58, *p* < 0.0001), no effect of genotype (*F*_1,15_ = 1.015, *p* = 0.3297) and no interaction genotype/time (*F*_2,26_ = 0.77, *p* = 0.475), *χ*^2^ ratio = 26.75, *df* = 1, *p* < 0.0001. Post hoc Sidak analysis showed differences between days 1–8 and 9–16, and 1–8 and 17–24 in WT and in KO. The REML analysis showed no differences in the 12-h period (time: *F*_1,10_ = 0.4520, *p* = 0.5166, genotype: *F*_1,12_ = 0.2203, *p* = 0.6472, interaction between time and genotype: *F*_1,10_ = 1.030, *p* = 0.344; *χ*^2^ ratio = 4.274, *df* = 1, *p* = 0.0393).

**Fig. 6:**
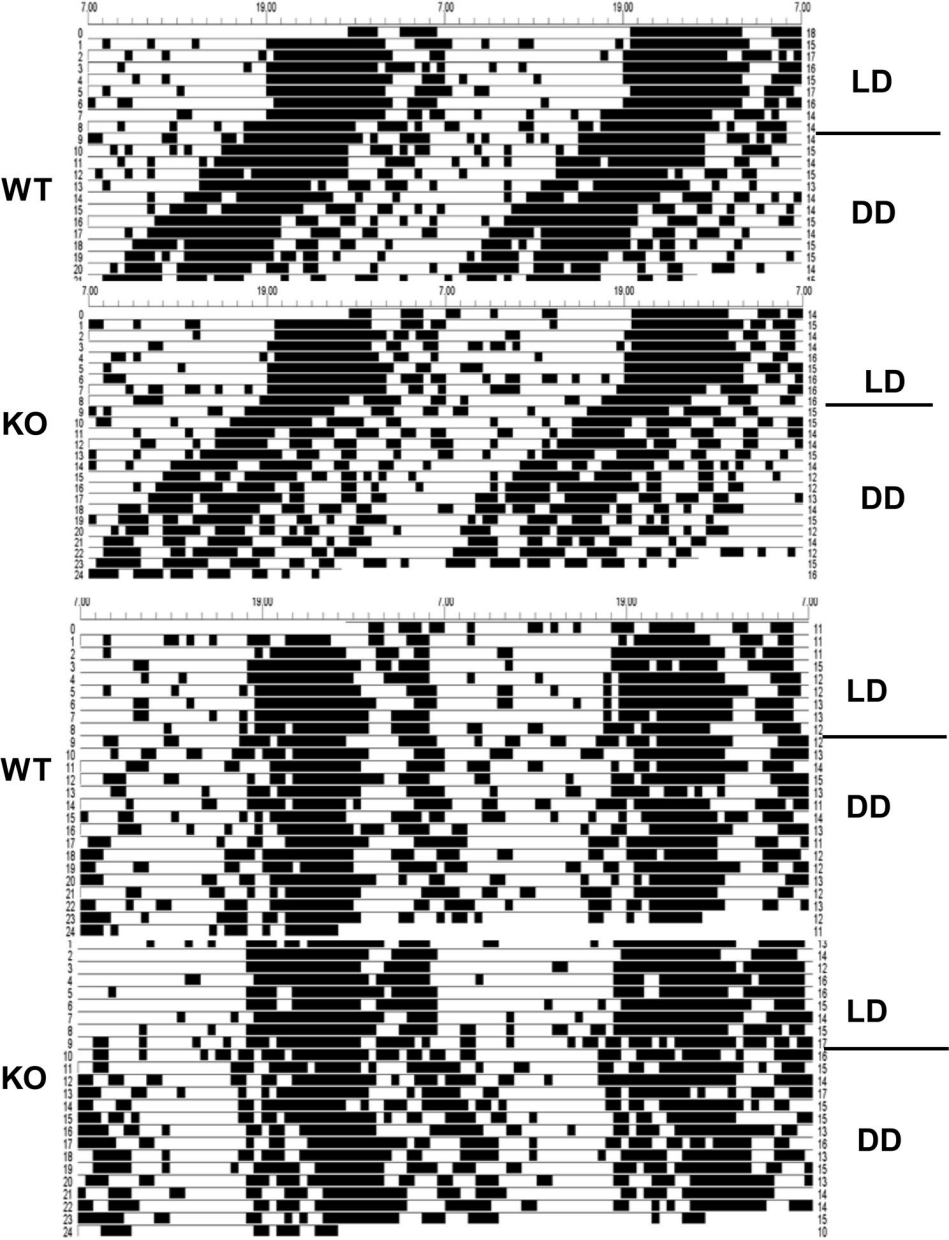
The sample actograms of WT and M_4_ KO mice. Top: the actograms in mice where the LD regime was switched on the 9th day to the DD regime. Bottom: the actograms in mice where the LD regime was switched on the 9th day to the DD regime and where the light pulse was applied on day 9. *WT* wild type mice, *KO* M_4_ KO mice, *LD* light/dark regime (see “Methods”). DD: dark/dark regime (see “Methods”)

The sample periodograms showing the time shift are shown in Fig. 6 (bottom).

### M_1_ MR autoradiography

**Fig. 7:**
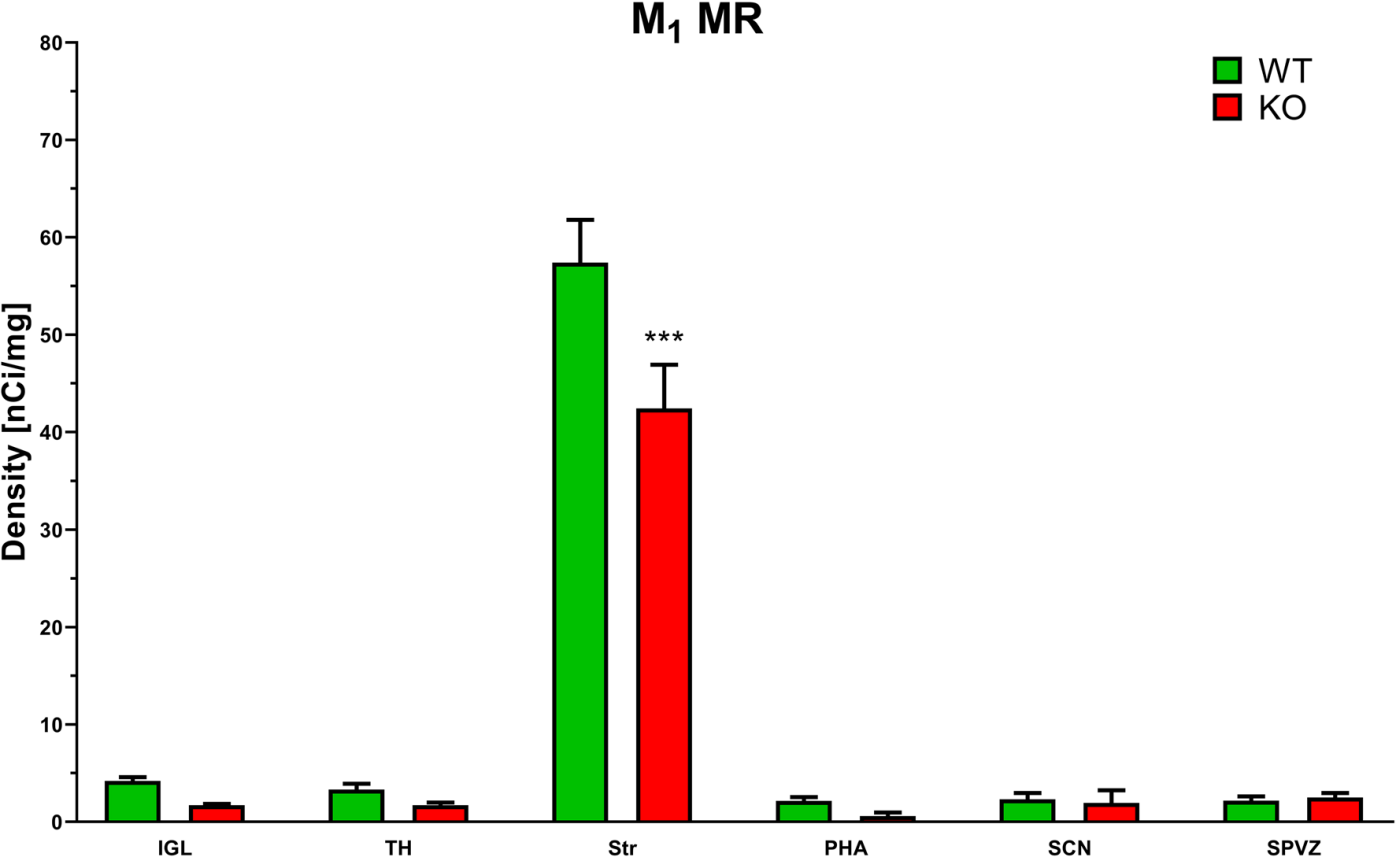
The densities of M_1_ MRs in the areas implicated in locomotor biological rhythm regulation. Ordinate: relative density (nCi/mg). Striatum (Str), thalamus (TH), suprachiasmatic nucleus (SCN), subparaventricular zone (SPVZ), posterior hypothalamic area (PHA), and intergeniculate leaflet (IGL). See legend for symbol explanation. ***p < 0.001, difference from WT animals

Two-way ANOVA showed a significant main effect of genotype (*F*_1,79_ = 6.378, *p* = 0.0135, *η*^2^ = 0.069) and area analyzed (*F*_5,79_ = 166.7, *p* < 0.001, *η*^2^ = 0.90) and a significant genotype–area interaction (*F*_5,73_ = 3.557, *p* = 0.0059, *η*^2^ = 0.019). Post hoc Sidak analysis showed differences between WT and KO M_1_ MR density in the striatum (see Fig. 7).

## Discussion

Here, we have shown that the biological rhythm activity curves differed after switching to the DD regime. These results suggest that the core clock output is changed. In the freely running clock condition (DD regime), the period (*τ*, duration of diurnal cycle) was shorter. This is in agreement with the Aschoff’s rule: Aschoff (Aschoff 1960) noted that under constant light conditions, the activity phase shortens in nocturnal organisms and lengthens in diurnal organisms.

Moreover, the day mean, night mean and their difference were higher in KO animals. Additionally, the mesor (midline value) was higher in KO animals than in WT. Therefore, KO animals retain increased locomotor activity in the active period in the freely running clock condition. There was no difference in the time in which the minimal slope occurred between WT and KO animals. The time difference, in which the maximal slope occurred, is questionable. However, our conclusion, that the core clock output is changed, is not affected by this finding. The periodograms showed differences between the LD and the DD regime both in WT and KO animals.

The light pulse applied on day 9 caused no differences in biological rhythm parameters between WT and KO animals. The periodograms showed differences between the LD and the DD regime both in WT and KO animals. This conclusion supports the hypotheses that the structures involved in biological rhythm regulation in WT and KO animals are most likely the same. Different conclusions were found in a paper in which the temperature rhythm was entrained faster than the locomotor rhythm after a light-phase shift (Satoh et al. 2006). These results suggested different intrinsic pacemaker regulation. However, in our previous work (Valuskova et al. 2018b), we did not find differences in temperature biological rhythm in M_4_ KO animals, which could be the reason for this difference.

In our previous work, we detected a significant density of M_4_ MRs in the motor cortex, striatum and intergeniculate leaflet. These densities, using recalculation (not used in the original paper) were: MoCx: 16.49 ± 2.12 nCi/mg, CPu: 52.38 ± 2.23 nCi/mg, IGL: 13.59 ± 1.58 nCi/mg. Thus, here we examined the amount of M_1_ MRs, another muscarinic receptor subtype implicated in locomotor coordination. The density of M_1_ MRs, as detected using the M_1_ MR-specific autoradiography protocol (Valuskova et al. 2018a), was not high in most brain areas implicated in locomotor activity biological rhythm changes. Very high amounts of M_1_ MRs were found in the striatum (relative density approximately 57 nCi/mg). The other areas revealed M_1_ MR densities between 2.1–4.2 nCi/mg. These densities were comparable to the low densities of M_4_ MRs in these areas. This is, according to our best knowledge, the first report about M_1_ and M_4_ MRs detection in the SCN, SPVZ, IGL, and PHA.

In Syrian hamsters, the application of the M_1_/M_4_ agonist (McN-A-343) directly to the SCN caused phase advances during the day (Basu et al. 2016). At first glance, this observation may be in disagreement with our results. However, M_4_ MRs are not present in the SCN (Valuskova et al. 2018b), and thus, these effects can be attributed to M_1_ MRs. M_1_ MRs are present in the SCN, although at a very low density. On the other hand, the SCN has a high density of cholinergic neurons (Abbott et al. 2013). In addition to that, (Yang et al. 2010) have demonstrated that carbachol (muscarinic non-specific agonist) induces hyperpolarization via background K^+^ currents. These effects were blocked by M_4_ antagonists and to a lesser degree by M_1_ antagonists.

In conclusion, there are three possibilities explaining this SCN-directed locomotor biological rhythm regulation: first, the biological rhythm is directed by M_1_ MRs, and the very low density is enough to regulate these events. Second, there is an interconnection between M_1_ MRs and M_4_ MRs that regulates biological rhythm. Third, structures other than the SCN are responsible for locomotor biological rhythm regulation. Our data on M_4_ and M_1_ density, however, very probably exclude the role of this muscarinic receptor subtype in SCN directed locomotor biological rhythm regulation what does not exclude its role in other SCN-mediated processes. Therefore, we suggest the third possibility as the most plausible.

Interestingly, WT female mice were shown to have a significantly larger phase response than estrogen receptor subtype 1 knock-out female mice during the early subjective night (Blattner and Mahoney 2013). This observation further supports the hypothesis regarding the connection between hormone levels and different types of locomotor activity as defined previously (Kuljis et al. 2013; Blizard et al. 1975; Morgan and Pfaff 2001; Ogawa et al. 2003).

The structures involved in locomotor biological rhythm regulation are, in many cases, the target or the source of acetylcholine action. The SCN is densely innervated by cholinergic neurons (Abbott et al. 2013). Muscarinic receptors have been shown to be involved in carbachol (muscarinic agonist)-induced phase shifts in circadian rhythms (Bina and Rusak 1996). However, our results suggest that structures other than the SCN are responsible for locomotor biological rhythm regulation. This suggestion is also in agreement with findings of the role of M_1_ MRs in SCN circadian rhythm (Liu and Gillette 1996; Gillette et al. 2001).

Cholinergic projections were also identified in the subparaventricular zone (Castillo-Ruiz and Nunez 2007). The density of M_1_ MRs in this area, as detected in our present study, was low, and there are no M_4_ MRs present. We also compared our results with in situ hybridization data in Allen Mouse Brain Atlas (Lein et al. 2007). However, it is necessary to stress that the amount of RNA does not necessarily correlate with the number of binding sites representing the real picture of receptors able to bind a specific ligand. For example, M_4_ MR showed an almost undetectable hybridization in all brain areas, but the amount of M_4_ MR binding in autoradiography was particularly high in the striatum. A relatively good correlation between in situ hybridization and autoradiography was found for M_1_ MR.

Cholinergic neurons have been shown to be present in the posterior hypothalamic area (Casini et al. 2018), although in carp (*Cyprinus carpio*). Our results showed MRs in the posterior hypothalamic area but no M_4_ MRs (Valuskova et al. 2018b), and the density of M_1_ MRs was low.

On the other hand, M_1_ MRs and M_4_ MRs are both present in the striatum (Felder et al. 2018), and both receptor types are important in locomotor regulation. In the thalamus, M_4_ MRs were shown to modulate glutamatergic transmission in the corticostriatal pathway (Pancani et al. 2014). In agreement with this finding, we found that approximately 50% of MRs in this region are M_4_ MRs (Valuskova et al. 2018b).

Taken together, our results suggest that the core clock output is changed in M_4_-deficient mice. The structures involved in biological rhythm regulation in WT and KO mice are most likely the same. The main area that affects the M_4_ MR-directed locomotor biological rhythm is likely the striatum together with coordinated interconnection with the thalamus and intergeniculate leaflet.

## References

[CR1] Abbott SM, Arnold JM, Chang Q, Miao H, Ota N, Cecala C, Gold PE, Sweedler JV, Gillette MU (2013). Signals from the brainstem sleep/wake centers regulate behavioral timing via the circadian clock. PLoS ONE.

[CR2] Abrahamson EE, Moore RY (2006). Lesions of suprachiasmatic nucleus efferents selectively affect rest-activity rhythm. Mol Cell Endocrinol.

[CR3] Aschoff J (1960). Exogenous and endogenous components in circadian rhythms. Cold Spring Harb Symp Quant Biol.

[CR4] Ballesta A, Innominato PF, Dallmann R, Rand DA, Lévi FA (2017). Systems chronotherapeutics. Pharmacol Rev.

[CR5] Basu P, Wensel AL, McKibbon R, Lefebvre N, Antle MC (2016). Activation of M1/4 receptors phase advances the hamster circadian clock during the day. Neurosci Lett.

[CR6] Bina KG, Rusak B (1996). Muscarinic receptors mediate carbachol-induced phase shifts of circadian activity rhythms in Syrian hamsters. Brain Res.

[CR7] Bina KG, Rusak B, Semba K (1993). Localization of cholinergic neurons in the forebrain and brainstem that project to the suprachiasmatic nucleus of the hypothalamus in rat. J Comp Neurol.

[CR8] Bina KG, Rusak B, Wilkinson M (1998). Daily variation of muscarinic receptors in visual cortex but not suprachiasmatic nucleus of Syrian hamsters. Brain Res.

[CR9] Blattner MS, Mahoney MM (2013). Photic phase-response curve in 2 strains of mice with impaired responsiveness to estrogens. J Biol Rhythms.

[CR10] Blizard DA, Lippman HR, Chen JJ (1975). Sex differences in open-field behavior in the rat: the inductive and activational role of gonadal hormones. Physiol Behav.

[CR11] Buchanan GF, Gillette MU (2005). New light on an old paradox: site-dependent effects of carbachol on circadian rhythms. Exp Neurol.

[CR12] Cain SW, Verwey M, Szybowska M, Ralph MR, Yeomans JS (2007). Carbachol injections into the intergeniculate leaflet induce nonphotic phase shifts. Brain Res.

[CR13] Casini A, Vaccaro R, Toni M, Cioni C (2018). Distribution of choline acetyltransferase (ChAT) immunoreactivity in the brain of the teleost *Cyprinus carpio*. Eur J Histochem EJH.

[CR14] Castillo-Ruiz A, Nunez AA (2007). Cholinergic projections to the suprachiasmatic nucleus and lower subparaventricular zone of diurnal and nocturnal rodents. Brain Res.

[CR15] Felder CC, Goldsmith PJ, Jackson K, Sanger HE, Evans DA, Mogg AJ, Broad LM (2018). Current status of muscarinic M1 and M4 receptors as drug targets for neurodegenerative diseases. Neuropharmacology.

[CR16] Fink-Jensen A, Schmidt LS, Dencker D, Schülein C, Wess J, Wörtwein G, Woldbye DPD (2011). Antipsychotic-induced catalepsy is attenuated in mice lacking the M4 muscarinic acetylcholine receptor. Eur J Pharmacol.

[CR17] Furukawa T, Murakami N, Takahashi K, Etoh T (1987). Effect of implantation of carbachol pellet near the suprachiasmatic nucleus on the free-running period of rat locomotor activity rhythm. Jpn J Physiol.

[CR18] Gannon RL, Millan MJ (2012). LY2033298, a positive allosteric modulator at muscarinic M4 receptors, enhances inhibition by oxotremorine of light-induced phase shifts in hamster circadian activity rhythms. Psychopharmacology.

[CR19] Gillette MU, Buchanan GF, Artinian L, Hamilton SE, Nathanson NM, Liu C (2001). Role of the M1 receptor in regulating circadian rhythms. Life Sci.

[CR20] Gomeza J, Zhang L, Kostenis E, Felder C, Bymaster F, Brodkin J, Shannon H, Xia B, Deng C-x, Wess J (1999). Enhancement of D1 dopamine receptor-mediated locomotor stimulation in M4 muscarinic acetylcholine receptor knockout mice. Proc Natl Acad Sci.

[CR21] Hughes ATL, Piggins HD (2012) Chapter 18—Feedback actions of locomotor activity to the circadian clock. In: Andries Kalsbeek MMTR, Russell GF (eds) Progress in brain research, vol 199. Elsevier, Amsterdam, pp 305–336. 10.1016/B978-0-444-59427-3.00018-6 10.1016/B978-0-444-59427-3.00018-6 22877673

[CR22] Hut RA, Van der Zee EA (2011). The cholinergic system, circadian rhythmicity, and time memory. Behav Brain Res.

[CR23] Ichikawa T, Hirata Y (1986). Organization of choline acetyltransferase-containing structures in the forebrain of the rat. J Neurosci.

[CR24] Kafka MS, Wirz-Justice A, Naber D, Moore RY, Benedito MA (1983). Circadian rhythms in rat brain neurotransmitter receptors. Fed Proc.

[CR25] Koshimizu H, Leiter L, Miyakawa T (2012). M4 muscarinic receptor knockout mice display abnormal social behavior and decreased prepulse inhibition. Mol Brain.

[CR26] Kuljis DA, Loh DH, Truong D, Vosko AM, Ong ML, McClusky R, Arnold AP, Colwell CS (2013). Gonadal- and sex-chromosome-dependent sex differences in the circadian system. Endocrinology.

[CR27] Lein ES, Hawrylycz MJ, Ao N, Ayres M, Bensinger A, Bernard A, Boe AF, Boguski MS, Brockway KS, Byrnes EJ, Chen L, Chen L, Chen T-M, Chi Chin M, Chong J, Crook BE, Czaplinska A, Dang CN, Datta S, Dee NR, Desaki AL, Desta T, Diep E, Dolbeare TA, Donelan MJ, Dong H-W, Dougherty JG, Duncan BJ, Ebbert AJ, Eichele G, Estin LK, Faber C, Facer BA, Fields R, Fischer SR, Fliss TP, Frensley C, Gates SN, Glattfelder KJ, Halverson KR, Hart MR, Hohmann JG, Howell MP, Jeung DP, Johnson RA, Karr PT, Kawal R, Kidney JM, Knapik RH, Kuan CL, Lake JH, Laramee AR, Larsen KD, Lau C, Lemon TA, Liang AJ, Liu Y, Luong LT, Michaels J, Morgan JJ, Morgan RJ, Mortrud MT, Mosqueda NF, Ng LL, Ng R, Orta GJ, Overly CC, Pak TH, Parry SE, Pathak SD, Pearson OC, Puchalski RB, Riley ZL, Rockett HR, Rowland SA, Royall JJ, Ruiz MJ, Sarno NR, Schaffnit K, Shapovalova NV, Sivisay T, Slaughterbeck CR, Smith SC, Smith KA, Smith BI, Sodt AJ, Stewart NN, Stumpf K-R, Sunkin SM, Sutram M, Tam A, Teemer CD, Thaller C, Thompson CL, Varnam LR, Visel A, Whitlock RM, Wohnoutka PE, Wolkey CK, Wong VY, Wood M, Yaylaoglu MB, Young RC, Youngstrom BL, Feng Yuan X, Zhang B, Zwingman TA, Jones AR (2007). Genome-wide atlas of gene expression in the adult mouse brain. Nature.

[CR28] Liu C, Gillette M (1996). Cholinergic regulation of the suprachiasmatic nucleus circadian rhythm via a muscarinic mechanism at night. J Neurosci.

[CR29] Morgan MA, Pfaff DW (2001). Effects of estrogen on activity and fear-related behaviors in mice. Horm Behav.

[CR30] Morin LP (2013). Neuroanatomy of the extended circadian rhythm system. Exp Neurol.

[CR31] Ogawa S, Chan J, Gustafsson J-A, Korach KS, Pfaff DW (2003). Estrogen increases locomotor activity in mice through estrogen receptor alpha: specificity for the type of activity. Endocrinology.

[CR32] Pancani T, Bolarinwa C, Smith Y, Lindsley CW, Conn PJ, Xiang Z (2014). M4 mAChR-mediated modulation of glutamatergic transmission at corticostriatal synapses. ACS Chem Neurosci.

[CR33] Paxinos G, Franklin KBJ (2008). The mouse brain in stereotaxic coordinates.

[CR34] Pekala D, Blasiak A, Lewandowski MH (2007). The influence of carbachol on glutamate-induced activity of the intergeniculate leaflet neurons—in vitro studies. Brain Res.

[CR35] Rusak B, Bina KG (1990). Neurotransmitters in the mammalian circadian system. Annu Rev Neurosci.

[CR36] Satoh Y, Kawai H, Kudo N, Kawashima Y, Mitsumoto A (2006). Temperature rhythm reentrains faster than locomotor rhythm after a light phase shift. Physiol Behav.

[CR37] Segal M, Dudai Y, Amsterdam A (1978). Distribution of an alpha-bungarotoxin-binding cholinergic nicotinic receptor in rat brain. Brain Res.

[CR38] Schmidt L, Thomsen M, Weikop P, Dencker D, Wess J, Woldbye DD, Wortwein G, Fink-Jensen A (2011). Increased cocaine self-administration in M4 muscarinic acetylcholine receptor knockout mice. Psychopharmacology.

[CR39] Silver J, Billiar RB (1976). An autoradiographic analysis of [3H]alpha-bungarotoxin distribution in the rat brain after intraventricular injection. J Cell Biol.

[CR40] Valuskova P, Farar V, Forczek S, Krizova I, Myslivecek J (2018). Autoradiography of 3H-pirenzepine and 3H-AFDX-384 in mouse brain regions: possible insights into M1, M2, and M4 muscarinic receptors distribution. Front Pharmacol.

[CR41] Valuskova P, Forczek ST, Farar V, Myslivecek J (2018). The deletion of M4 muscarinic receptors increases motor activity in females in the dark phase. Brain Behav.

[CR42] Valuskova P, Riljak V, Forczek ST (2019). Variability in the drug response of M4 muscarinic receptor knockout mice during day and night time. Front Pharmacol.

[CR43] van den Pol AN, Tsujimoto KL (1985). Neurotransmitters of the hypothalamic suprachiasmatic nucleus: immunocytochemical analysis of 25 neuronal antigens. Neuroscience.

[CR44] van der Zee EA, Streefland C, Strosberg AD, Schroder H, Luiten PG (1991). Colocalization of muscarinic and nicotinic receptors in cholinoceptive neurons of the suprachiasmatic region in young and aged rats. Brain Res.

[CR45] Wirz-Justice A (1987). Circadian rhythms in mammalian neurotransmitter receptors. Prog Neurobiol.

[CR46] Woolley ML, Carter HJ, Gartlon JE, Watson JM, Dawson LA (2009). Attenuation of amphetamine-induced activity by the non-selective muscarinic receptor agonist, xanomeline, is absent in muscarinic M4 receptor knockout mice and attenuated in muscarinic M1 receptor knockout mice. Eur J Pharmacol.

[CR47] Wyartt C (2018). Taking a big step towards understanding locomotion. Trends Neurosci.

[CR48] Yang J-J, Wang Y-T, Cheng P-C, Kuo Y-J, Huang R-C (2010). Cholinergic modulation of neuronal excitability in the rat suprachiasmatic nucleus. J Neurophysiol.

[CR49] Zatz M (1979). Photoentrainment, pharmacology, and phase shifts of the circadian rhythm in the rat pineal. Fed Proc.

[CR50] Zatz M, Brownstein MJ (1979). Intraventricular carbachol mimics the effects of light on the circadian rhythm in the rat pineal gland. Science.

